# Salivary markers of oxidative stress in oral diseases

**DOI:** 10.3389/fcimb.2015.00073

**Published:** 2015-10-20

**Authors:** L'ubomíra Tóthová, Natália Kamodyová, Tomáš Červenka, Peter Celec

**Affiliations:** ^1^Institute of Molecular Biomedicine, Faculty of Medicine, Comenius UniversityBratislava, Slovakia; ^2^Center for Molecular Medicine, Slovak Academy of SciencesBratislava, Slovakia; ^3^Department of Molecular Biology, Faculty of Natural Sciences, Comenius UniversityBratislava, Slovakia; ^4^Institute of Pathophysiology, Faculty of Medicine, Comenius UniversityBratislava, Slovakia

**Keywords:** saliva, biomarkers, oxidative stress, antioxidant status, oral diseases

## Abstract

Saliva is an interesting alternative diagnostic body fluid with several specific advantages over blood. These include non-invasive and easy collection and related possibility to do repeated sampling. One of the obstacles that hinders the wider use of saliva for diagnosis and monitoring of systemic diseases is its composition, which is affected by local oral status. However, this issue makes saliva very interesting for clinical biochemistry of oral diseases. Periodontitis, caries, oral precancerosis, and other local oral pathologies are associated with oxidative stress. Several markers of lipid peroxidation, protein oxidation and DNA damage induced by reactive oxygen species can be measured in saliva. Clinical studies have shown an association with oral pathologies at least for some of the established salivary markers of oxidative stress. This association is currently limited to the population level and none of the widely used markers can be applied for individual diagnostics. Oxidative stress seems to be of local oral origin, but it is currently unclear whether it is caused by an overproduction of reactive oxygen species due to inflammation or by the lack of antioxidants. Interventional studies, both, in experimental animals as well as humans indicate that antioxidant treatment could prevent or slow-down the progress of periodontitis. This makes the potential clinical use of salivary markers of oxidative stress even more attractive. This review summarizes basic information on the most commonly used salivary markers of oxidative damage, antioxidant status, and carbonyl stress and the studies analyzing these markers in patients with caries or periodontitis.

## Introduction

Saliva has become a popular diagnostic fluid for research and clinics in recent years. Its availability, easy collection and possibility of repeated non-invasive sampling makes it ideal for screening, diagnosis, or monitoring of many diseases. However, many technical issues have to be overcome and sensitivity as well as specificity have to be increased before routine clinical use. Several articles have been published dealing with saliva and its diagnostic potential in the past (Kaufman and Lamster, 2002; Chiappin et al., 2007; Lee and Wong, 2009). One review article has summarized the literature on oxidative stress in oral cavity-related pathologies. However, the review was not specifically oriented on oxidative stress in saliva (Iannitti et al., 2012). One critical review on this topic has been published recently, but was mainly focused on the methodology and statistical analysis used in the published studies (Wang et al., 2015a).

Periodontitis and dental caries represent the most common oral diseases. Very often, low adherence to oral hygiene and follow-up treatment lead to worsening of the disease and subsequently to loss of teeth (Renz et al., 2007). A non-invasive approach to diagnose and to monitor the progress of periodontitis and dental caries is needed that would improve the adherence and the overall therapeutic outcome. This review therefore aims to summarize the current findings in the research of oxidative stress analyzed in saliva in relation to oral diseases.

## Saliva as a diagnostic fluid

Saliva is produced by secretion from the three major salivary glands (the parotid, submandibular, and sublingual glands) and numerous minor salivary glands. The collected whole saliva is a more complex mixture of fluids including gingival cevicular fluid, as well as oral, nasal, and mucosal transudate (Humphrey and Williamson, 2001). Additionally, oral bacteria and their metabolites, desquamated epithelial and blood cells, food debris and various chemical products are present in the saliva (de Almeida Pdel et al., 2008). Daily saliva production is estimated to be between 0.75 and 1.5 L in healthy adults. Salivary secretion is under both neural and hormonal control (Proctor and Carpenter, 2007). Nevertheless, salivary flow rate can be affected by various factors, such as circadian cycle, age, hydratation, chewing, oral hygiene, physical exercise, and others (Dawes, 1972; Chicharro et al., 1998; Chiappin et al., 2007; de Almeida Pdel et al., 2008).

Physiologial pH of saliva is between 6.2 and 7.4 (Schipper et al., 2007). From biochemical point of view, saliva is an aqueous solution (more than 99% is water) containing numerous organic and anorganic molecules (Greabu et al., 2009; Lima et al., 2010). Saliva may reflect the current physiological condition of the body and therefore is often called “the mirror of health of the organism” (Farnaud et al., 2010; Yoshizawa et al., 2013). The exchange between plasma and saliva takes place in the salivary ducts, which are separated from the circulation system as a thin layer of epithelial cells. The exchange includes active transport, diffusion across the cell membrane by passive diffusion directed by the concentration gradient (Lee and Wong, 2009). In patients suffering from oral diseases such as periodontitis there is a higher probability of blood leakage into saliva. This leads to the occurrence of blood components in saliva (Schwartz and Granger, 2004). This could interfere with various analytical methods and, thus, hinder the diagnostic use of saliva. However, at least for some of the most commonly used markers of oxidative stress we have found that blood contamination up to 1% does not affect even spectrophotometric methods and samples with higher blood concentrations can easily be excluded (Kamodyová et al., 2015). Since saliva represents a rapidly changing dynamic environment, it can potentially be used for long-term monitoring of oral diseases. In addition, new high-throughput approaches are being introduced for fast, reliable, and reproducible salivary diagnostic tests. Research on saliva cannot escape the current technological revolution. The whole salivary metabolome has already been described using several methodological approaches (Dame et al., 2015).

Similarly to plasma and tissues, free radicals and reactive oxygen/nitrogen species (ROS/RNS) in saliva play an important role in redox-dependent signaling and are necessary for physiological functions (Valko et al., 2007). On the other hand, excessive production of free radicals can lead to oxidative stress (Sies, 1997). Redox balance is than shifted in favor of oxidants. ROS/RNS can induce oxidative damage to cellular components with serious pathophysiological consequences (Devasagayam et al., 2004). On contrary, various antioxidant mechanisms are present in saliva including low molecular antioxidants—glutathione, ascorbic, and uric acid as well as melatonin (Moore et al., 1994; Balaji et al., 2015). Antioxidant enzymes such as superoxide dismutase, catalase, and glutathione peroxidase are present in saliva (Battino et al., 2002). Their function is to protect oral cavity against the negative effects of endogenous and exogenous ROS/RNS. Additionally, membrane and DNA repair enzymes as well as proteases that degrade oxidatively modified proteins reduce the consequences of oxidative damage in saliva. Saliva is intended to be the first line defense against free radicals (Amerongen and Veerman, 2002; Battino et al., 2002). The dysbalance between the production of free radicals and antioxidant status in favor of oxidants is called oxidative stress. Oxidative stress has been implicated in the etiology and pathogenesis of several oral diseases including dental caries and periodontitis (Iannitti et al., 2012). This has already been shown by expression analysis of antioxidant genes in periodontitis patients (Zeidan-Chulia et al., 2013). Although it is not clear whether it is a cause or consequence of the disease process.

## Salivary biomarkers of oxidative, carbonyl stress, and antioxidant status

Biomarkers are any characteristics which can be objectively measured and allow to predict the diagnosis, onset, or progression of a disease (Maiese et al., 2010). Optimal biomarkers for diagnostics of oxidative stress related diseases should be stable, accumulated in detectable concentrations, reflect specific oxidation pathways, and correlate with disease severity (Dalle-Donne et al., 2006). Considering free radicals are highly reactive and have a short half-life, the products formed from the reaction of ROS/RNS with cellular macromolecules are used preferentially as biomarkers of oxidative damage (Palmieri and Sblendorio, 2007a,b). Lipid peroxidation products, oxidized proteins, and products of DNA oxidation and fragmentation—are used for the assessment of oxidative stress. Measurement of various antioxidants or total antioxidant status represent another option of analyzing the redox status. Nevertheless, the use of a panel of biomarkers instead of a single parameter provides more informative results, reduces false positive and false negative results and enables a better understanding of the underlying pathomechanisms. The recent technological advances enabled the progress in systems biology and saliva research is moving into the omics world. Also using the metabolomic approach markers of oxidative stress were found to be among the discriminative between patients with periodontitis and healthy controls (Barnes et al., 2014). This confirms that the research of salivary markers of oxidative stress is worth the effort.

### Lipid peroxidation

Lipid peroxidation is a reaction of lipids such as polyunsaturated fatty acids with ROS/RNS leading to formation of lipid hydroperoxides. This is accompanied by a complex process of degradation and decomposition reactions of hydroperoxides, whereby a wide range of products is formed (Dotan et al., 2004; Palmieri and Sblendorio, 2007a). The endproducts of lipid peroxidation are more stable than free radicals. Additionally, they can further react with other macromolecules, including DNA, proteins and phospholipids, far from the site of their production (Dalle-Donne et al., 2006). The most studied marker of lipid peroxidation is malondialdehyde (MDA). MDA is produced from fatty acids with two or more methylene-interrupted double bonds (Ayala et al., 2014). Standard method used for MDA detection is the spectrophotometric assay developed by Yagi, which is based on the reaction with thiobarbituric acid in acidic environment. It is also referred to as thiobarbituric acid reacting substances (TBARS) assay (Yagi, 1976). This assay is not specific for MDA and other aldehydes may react with thiobarbituric acid to produce a compound that absorbs wavelengths in the same range as MDA. Liquid chromatography or mass spectroscopy methods were reported as more reliable and specific for the measurement of MDA (Akalin et al., 2007), but the TBARS assay still represents a commonly used, cheap and high throughput method for the quantitative analysis of lipid peroxidation.

Another marker produced during lipid peroxidation is 4-hydroxy-2-nonenal that is generated by free radical attack on ω—6 polyunsaturated fatty acids (arachidonic, linoleic, and linolenic acids) (Sayre et al., 2006). Isoprostanes are unique products of lipid peroxidation of arachidonic acid and are considered to be reliable biomarkers of free radical mediated lipid peroxidation *in vivo* (Devasagayam et al., 2004; Spickett, 2013). Conjugated dienes are another option for analysis of lipid peroxidation. Unfortunately, few studies dealt with several of these markers, so the data about their usefulness for oral disease monitoring are limited.

### Protein oxidation

Proteins are major targets for ROS/RNS because they are highly abundant and are responsible for most functional processes in the cell (Dalle-Donne et al., 2003). The oxidation of proteins can take place at the level of single amino acid residues, it can lead to fragmentation of polypeptide chains or to covalent cross-linking of two amino acids either of the same or of two different proteins (Shacter, 2000). Oxidized proteins are either catabolized in proteosomal and lysosomal pathways or aggregated and accumulated in cellular compartments (Stadtman and Berlett, 1998).

Widespread methods for assessment of protein oxidation are the measurement of carbonyl groups by specific antibodies in ELISA or Western blot and by spectrophotometric assay based on dinitrophenylhydrazine derivitization (Dalle-Donne et al., 2003; Cabiscol et al., 2014). The advantages of protein carbonyls as a marker in comparison to lipid peroxidation products include early production and greater stability of oxidized proteins. Nonetheless, the production of protein carbonyl groups can be induced by almost all types of ROS and so, the protein carbonyl assay does not provide information about the source of oxidative stress. A major factors influencing most biomarkers of oxidative stress and protein carbonyl especially is aging. The relatively high correlation coefficients between salivary carbonyls and age led to a suggestion of protein carbonyls as an alternative biomarker of aging (Wang et al., 2015b).

Advanced oxidation protein products (AOPP) represent a sensitive biomarker of protein oxidation, especially due to neutrophil activation and the enzymatic activity of myeloperoxidase (Witko-Sarsat et al., 1996). AOPP were formerly thought to be a novel uremic toxin, reflecting highly oxidized protein status, but oxidized fibrinogen was found to be the major molecule responsible for the increase of the AOPP concentration (Selmeci, 2011). This should be taken into account, when interpreting the results.

### Oxidative DNA damage

The ROS/RNS react with DNA inducing damage to purine and pyrimidine bases and also the deoxyribose backbone (Halliwell, 2000). Pyrimidine damage products include thymine glycol, uracil glycol, urea residue, 5-hydroxydeoxyuridine, 5-hydroxydeoxycytidine, hydantoin, and others. Purine damage products are 8-hydroxydeoxyguanosine (8-OHdG), 8-hydroxydeoxyadenosine, formamidopyrimidines, and other less characterized purine oxidative products (Cadet et al., 2003).

Measurement of 8-OHdG has been used to assess “whole-body” oxidative DNA damage using various analytical methods (Henderson et al., 2010). The issue with measuring 8-OHdG concentration is that 8-OHdG may not truly reflect oxidative damage to DNA. 8-OHdG can arise not just from removal of oxidized guanine residues from DNA by repair processes but also from degradation of oxidized dGTP in the DNA precursor pool. Recently published data, however, show that there is a clear strong association between the salivary concentrations of 8-OHdG and the clinical indices of periodontal status (Villa-Correa et al., 2015).

### Carbonyl stress

Carbohydrates such as glucose react non-enzymatically with the free amino groups of proteins and proceeds from reversible Schiff base adducts to more stable Amadori products. Some Amadori products are further converted to advanced glycation end products (AGEs) through a series of chemical rearrangements, dehydration, and fragmentation reactions (Ott et al., 2014). In addition to endogenously formed, AGEs can also be derived from exogenous sources such as smoking and food (Singh et al., 2001; Nass et al., 2007). Carbonyl precursors can form oxidative AGEs such as N^ε^-(carboxymethyl)lysine and pentosidine or non-oxidative AGEs such as 3-deoxyglucosone or methylglyoxal (Singh et al., 2001). Pentosidine is derived exclusively from carbohydrate-derived carbonyl group and protein amino group while N^ε^-(carboxymethyl)lysine originates not only from carbohydrates but also from autooxidation of lipids and amino acids (Zoccali et al., 2000). N^ε^-(carboxymethyl)lysine therefore serves as a general biomarker of oxidative stress, mostly assessed by ELISA or Western blotting.

AGEs are a heterogeneous group of protein-bound moieties and are characterized by browning, fluorescence, and crosslinking. Their determination is based on detection of specific fluorescence of AGEs at 370 nm excitation and 440 nm emission (Münch et al., 1997). Other methods such as ELISA, polyclonal antibodies, HPLC, or immunohistochemistry have been also applied to determine specific AGEs (Ahmed et al., 2002; Lapolla et al., 2005; Schmitt et al., 2005). Measurement of non-enzymatically glycated total proteins is referred to as fructosamine assay (Armbruster, 1987). Glucose bound to protein by aldimine linkage undergoes Amadori rearrangement through nonenzymatic glycation to the ketoamine (generically termed fructosamine). Fructosamine spectrophotometric assay is a simple test based upon the property of fructosamines to act as reducing agents in alkaline solution (San-Gil et al., 1985).

### Nitrosative stress

Similarly to oxidative stress, nitrosative stress is characterized as the imbalance between reactive nitrogen species and the antioxidants in favor of pro-oxidant RNS. Immoderate/excessive production of nitric oxide (NO) have detrimental effects on biomacromolecules. Due to its highly reactive nature, NO reacts with other reactive species (i.e., superoxide) leading to formation of more reactive compounds resulting in cytotoxic effects (Eiserich et al., 1998). On the other hand, NO can be oxidized to nitrite (NO${}_{2}^{-}$) and subsequently oxidized to nitrate (NO${}_{3}^{-}$). Therefore, the total concentrations of nitrite and nitrate are summed to adequately determine complex NO production. Salivary nitrates/nitrites are measured by Gries colorimetric assay (Andrukhov et al., 2013). Salivary NO and its derivates have been studied in relation to plasma NO, endothelial function, dietary intake of NO donors etc. However, most of the analyzed correlations were not significant (Clodfelter et al., 2015). Although the test was originally developed to help cardiologists, it is clear that the salivary NO concentrations are most affected by local oral processes and could, thus, be more usable for dentists and their patients.

### Antioxidant status

The measurement of individual antioxidants in biological samples is time consuming, labor-intensive, costly and requires complicated chemical techniques. In addition, the antioxidant effects are additive and therefore total antioxidant capacity of samples is preferentially measured (Erel, 2004). Antioxidant capacity assays can be divided into assays involving oxidants that are not necessarily pro-oxidants and assays involving oxidants that are pro-oxidants (Prior and Cao, 1999). To the first group of assays belong for example the ferric reducing/antioxidant power (FRAP) assay and the Trolox equivalent antioxidant capacity. The second group of assays includes total radical trapping parameter assay, luminol-based assays, dichlorofluorescin-diacetate based assay, crocin based assays, phycoerythrin based assays, and oxygen radical absorbance capacity assay (Prior and Cao, 1999). The increased antioxidant capacity can be a consequence of an adaptive response to a long term increased oxidative stress. On the other hand, the decrease in antioxidant capacity is not necessarily an undesirable condition when the production of reactive species decreases. The results of antioxidant assays should therefore be interpreted with caution.

## Salivary oxidative stress in oral diseases

### Dental caries

Dental caries is the most prevalent oral disease worldwide affecting both, children and adults and leading to pain and tooth loss. Systematic review dealing with the pathogenesis, epidemiology, diagnosis, and treatment of dental caries was published previously (Selwitz et al., 2007). Dental caries is a multifactorial inflammatory disease. However, the primary trigger is usually acidic by-products formed during bacterial fermentation of carbohydrates (Selwitz et al., 2007). In a recent review, the concept of inflammatory response in dentin, tightly connected with oxidative stress leading to the destruction of dental hard tissues is discussed (Southward, 2011). It was shown that dentinal fluid movement is inhibited by high sucrose levels causing down-regulation of parotid hormone from hypothalamic signaling. The consequence is that teeth become susceptible to bacterial acids (Leonora et al., 1993). The important role of ROS and antioxidants on the regulation of parotid hormone was hypothesized in a review dealing with the systemic theory of dental caries (Southward, 2011).

The importance of saliva in terms of salivary flow and antibacterial protection is generally accepted for the pathogenesis of dental caries (Lenander-Lumikari and Loimaranta, 2000; Stookey, 2008). The role of oxidative stress in dental caries is less clear, but it is the focus of intense research. Nine studies performed on children and four studies performed on adult subjects analyzing salivary oxidative stress in relation to dental caries are summarized in Table 1. Most research dealing with salivary oxidative stress in relation to dental caries is analyzing the antioxidant properties of saliva. Tulunoglu et al. have measured the antioxidant capacity of saliva in 80 children with and without caries activity (Tulunoglu et al., 2006). Children were divided into 8 groups according to age, gender, and caries activity. Non-significantly higher TAC was observed in caries active children and this observation was attributed to higher protein concentrations in caries active children. The exception was the group of 11–15 years old girls, where total protein and antioxidant capacity were lower in caries active group (Tulunoglu et al., 2006). In a very similar study TAC was analyzed in 120 children with and without caries activity (Preethi et al., 2010). Similarly to the previous study, children were divided into 8 groups. TAC was significantly higher in caries active girls and also boys in comparison to age matched control subjects. Total proteins were significantly higher in caries active in comparison to caries free children (Preethi et al., 2010; Dodwad et al., 2011). In the next study, relationship between the total antioxidant capacity of saliva and dental caries was assessed in deciduous and permanent teeth of Saharan children (Uberos et al., 2008). This study is one of the largest focusing on caries and salivary markers of antioxidant status. The authors have found a higher TAC in deciduous teeth of caries active patients than of caries free patients. Linear association was observed between the number of deciduous, but not permanent teeth affected by caries and TAC (Uberos et al., 2008). Hegde et al. and Kumar et al. have found that TAC in children with early childhood caries is higher than in controls with age being a significant confounding factor (Hegde et al., 2009; Kumar et al., 2011). The results were confirmed in another study in very young children (Muchandi et al., 2015). In a case-control study performed on 80 children FRAP as a measure of antioxidant status higher in children with caries (Mahjoub et al., 2014). Similarly, TAC was higher in adolescent males with active caries (Ahmadi-Motamayel et al., 2013). The reason for the higher antioxidant status in all studies remains unclear. A likely mechanism could include the response of the host to an infection/inflammation. All above mentioned studies had a case-control design. The study conducted by Tóthová et al. had a cross-sectional design with 82 children (4–18 years old) (Tóthová et al., 2013a). In this study beyond salivary antioxidant status (TAC and FRAP) also oxidative stress markers were analyzed in relation to caries index in children. These included TBARS, AOPP, and AGEs. Multivariate analysis showed that salivary AOPP are related to caries index (eta 8.6%), however ANOVA revealed no significant association between CI and AOPP (Tóthová et al., 2013a).

**Table 1:** **Association studies investigating salivary markers of oxidative stress and dental caries**.

**Patients**	**Analyzed markers and methods**	**Detected concentrations**	**Results**	**Ref**.
82 consecutive pediatric dental patients	AOPP (SPH), TBARS (SFL), AGES (SFL), FRAP (SPH), TAC (SPH)	Children with CI: 0, AOPP (160 μmol/L^#^), TBARS (0.029 μmol/L^#^), AGES (1 g/L^#^), FRAP (80 μmol/L^#^), TAC (1.6 mmol/L^#^) Children with CI: 1, AOPP (125 μmol/L^#^), TBARS (0.035 μmol/L^#^), AGES (0.95 g/L^#^), FRAP (68 μmol/L^#^), TAC (1.55 mmol/L^#^); Children with CI: 2, AOPP (130 μmol/L^#^), TBARS (0.034 μmol/L^#^), AGES (0.85 g/L^#^), FRAP (68 μmol/L^#^), TAC (1.58 mmol/L^#^)	AOPP were related to CI (eta 8.6%, *p* < 0.03) in general linear model	Tóthová et al., 2013a
50 children with severe early childhood caries (S-ECC), 50 healthy control children	TAC (SPH)	Control children, TAC (0.568 ± 0.169 mmol/L); Children with caries, TAC (1.729 ± 0.297 mmol/L)	↑ TAC in children with S-ECC (*p* < 0.001), linear regression between TAC and DMFT score (*p* < 0.001)	Kumar et al., 2011
80 children, 8 groups—according to age, gender and caries activity (*n* = 10/per group)	TAC (SPH)	Girls, age 7–10, caries free, TAC (0.39 ± 0.08 mmol/L); Boys, age 7–10, caries free, TAC (0.48 ± 0.20 mmol/L); Girls, age 11–15, caries free, TAC (0.65 ± 0.16 mmol/L); Boys, age 11–15, caries free, TAC (0.56 ± 0.30 mmol/L); Girls, age 7–10, caries active, TAC (0.45 ± 0.13 mmol/L); Boys, age7–10, caries active, TAC (0.60 ± 0.15 mmol/L); Girls, age 11–15, caries active, TAC (0.57 ± 0.18 mmol/L); Boys, age 11–15, caries active, TAC (0.69 ± 0.25 mmol/L)	↑ TAC in caries active groups (*p* = ns) except the 11 to 15-year-old girls group (*p* = ns)	Tulunoglu et al., 2006
120 children, 8 groups—according to age, gender and caries activity (*n* = 15/per group)	TAC (SPH)	Girls, age 7–10, caries free, TAC (0.16 ± 0.03 μmol/L); Boys, age 7–10, caries free, TAC (0.16 ± 0.05 μmol/L)g; Girls, age 11–14, caries free, TAC (0.19 ± 0.04 μmol/L); Boys, age 11–14, caries free, TAC (0.19 ± 0.04 μmol/L); Girls, age 7–10, caries active, TAC (0.23 ± 0.05 μmol/L); Boys, age7–10, caries active, TAC (0.20 ± 0.04 μmol/L); Girls, age 11–14, caries active, TAC (0.23 ± 0.04 μmol/L); Boys, age 11–14, caries active, TAC (0.22 ± 0.04 μmol/L)	↑ TAC in children with caries in comparison to children without caries (*p* < 0.05)	Preethi et al., 2010
126 children (4.5–14.5 years), caries in deciduous teeth (78.6% of children), caries in permanent teeth (77.8% of children)	TAC (SPH)	Caries free in deciduous teeth, TAC (7.8 ± 4.0 1/IC50); caries free in permanent teeth, TAC (7.8 ± 5.0 1/IC50); caries active in deciduous teeth, TAC (10.6 ± 11.1 1/IC50); caries active in permanent teeth, TAC (9.0 ± 8.0 1/IC50) Caries free group, TAC (7.3 ± 4.3 1/IC50); Group with caries activity 1–2, TAC (7.2 ± 4.2 1/IC50); Group with caries activity 3–4, TAC (8.7 ± 4.7 1/IC50); Group with caries activity 5+, TAC (11.5 ± 12.4 1/IC50)	↑ TAC in patients with caries in deciduous teeth than among those without caries (p=0.06); linear regression between the number of deciduous teeth affected by caries and TAC (*p* = 0.004)	Uberos et al., 2008
100 children, 4 groups—with early childhood caries and controls (below 71 months), with rampant caries and controls (6–12 years)	TAC (SPH)	Early childhood caries TAC (25.58 ± 12.12 mmol/l); Early childhood caries free controls, TAC (14.15 ± 4.24 mmol/l) Rampant caries, TAC (46.12 ± 0.99 mmol/l); Rampant caries free controls, TAC (22.96 ± 4.76 mmol/l)	↑ TAC in children with caries (*p* < 0.001), TAC was increased with the age of the children (*p* < 0.001)	Hegde et al., 2009
80 children, (aged 3–5 years), 2 groups—with severe early childhood caries and controls (*n* = 40/per group)	FRAP (SPH)	N/A	↑ FRAP in children with severe early childhood caries (*p* = 0.025)	Mahjoub et al., 2014
50 children, (aged 3–5 years), 2 groups—with severe early childhood caries and controls (*n* = 25/per group)	TAC (SPH)	Severe early caries, TAC (1.82 ± 0.19 mmol/l saliva); Control group TAC (1.08 ± 0.17 mmol/l saliva)	↑ TAC in children with severe early childhood caries (*p* < 0.0001)	Muchandi et al., 2015
100 healthy high school students (age 15–17 years), 4 groups according to gender and caries activity. (Caries active was confirmed when volunteer had at least 5 caries surfaces)	TAC (SPH)	Boys, caries active TAC (59.72 ± 12.15); Boys, caries free TAC (41.32 ± 9.92); Girls, caries active TAC(40.95 ± 15.48); Girls, caries free TAC (40.16 ± 11.88)	↑ TAC in caries active males when compared to caries free males (*p* < 0.001). In females, no difference in TAC (*p* < 0.84). ↑ TAC in males when compared to females (*p* < 0.001)	Ahmadi-Motamayel et al., 2013
21 adult patients with caries, 16 controls	GSH (SPH), LPO (SPH)	Caries group, GSH (1.6 ± 0.75 mg/g protein), LPO (0.3 ± 0.15 μmol MDA/g protein); Control group, GSH (2.2 ± 0.8 mg/g protein), LPO (0.33 ± 0.28 μmol MDA/g protein)	↓ GSH in caries group (*p* < 0.05), negative correlation between DMFT and GSH (*p* < 0.05)	Oztürk et al., 2008
67 adult patients with dental caries, 50 controls	TBARS (SPH)	Females, caries free, MDA (3.24 ± 0.54 ng/mL); Males, caries free, MDA (3.38 ± 0.33 ng/mL); Females, caries active, MDA (3.36 ± 1.42 ng/mL); Males, caries active, MDA (3.42 ± 0.22 ng/mL)	↑ MDA in caries active patients (*p* = ns)	Rai et al., 2006
100 adult patients, 4 groups (*n* = 25/per group)—control group, group I (DMFT < 3), group II (DMFT < 10), group III (DMFT > 10)	TAC (SPH)	Control group, TAC (0.34 ± 0.13 μmol/L); Group I, TAC (0.44 ± 0.13 μmol/L); Group II, TAC (0.56 ± 0.26 μmol/L); Group III, TAC (0.59 ± 0.18 μmol/L)	↑ TAC was observed in groups with higher DMFT score (*p* < 0.001)	Hegde et al., 2013

TAC, total antioxidant capacity; AOPP, advanced oxidation protein products; TBARS, thiobarbituric acid reacting substances; AGES, advanced glycation end products; FRAP, ferric reducing antioxidant power; GSH, reduced glutathione; LPO, lipid peroxidation; SPH, spectrophotometry; SFL, spectrofluorometry;

# *denotes levels estimated from graph*.

The study conducted in adults by Öztürk et al. was the first study analyzing the role of salivary glutathione (GSH) as an antioxidant in relation to dental caries (Oztürk et al., 2008). Significantly lower GSH concentrations were detected in adults with caries compared to subjects without caries and a negative correlation was observed between clinical indices and GSH. In this study no difference in lipid peroxidation was observed between subjects with and without caries (Oztürk et al., 2008). Rai et al. have analyzed TBARS in the saliva of caries active patients. Higher lipid peroxidation was detected in caries active patients than in control patients (Rai et al., 2006). Ahmadi-Motamayel et al. have analyzed TAC in caries active and caries free students (Ahmadi-Motamayel et al., 2013). In this study higher TAC was found in the group with caries, especially in males. Subtle gender differences were indicated by lower TAC in caries active females than in caries active males (Ahmadi-Motamayel et al., 2013). The association between TAC and caries severity was confirmed later (Hegde et al., 2013).

Most of the studies found that the antioxidant status is higher in caries active probands. As emphasized by Prior and Cao, an increased antioxidant capacity may be an adaptive response to increased oxidative stress (Prior and Cao, 1999). On the other hand, decreased GSH levels could suggest a tendency to decreased antioxidant status (Oztürk et al., 2008). The contradictory findings could be partially explained by temporal differences. As an acute effect the antioxidant status could be increased, but long term effects could contribute to consumption of antioxidants. It is clear that a panel of oxidative stress and antioxidant status markers is needed to interpret the role of oxidative stress in disease pathogenesis and progression (Prior and Cao, 1999). The only study in which a panel of salivary oxidative stress and antioxidant status markers was analyzed was the study conducted in children by Tóthová et al. (2013a). Although association studies have a clear outcome, there is a lack of mechanistic studies on the role of ROS or oxidative damage in saliva in relation to dental caries. Based on previous studies, it appears that there is no association between salivary lipid peroxidation and dental caries (Rai et al., 2006; Oztürk et al., 2008; Tóthová et al., 2013a). However, to interpret the relationship between dental caries and oxidative stress in saliva it is necessary to perform more carefully designed studies incorporating whole palette of oxidative stress and antioxidant status markers and, more importantly, experimental animal studies focusing on the underlying mechanism associated with ROS/RNS.

### Periodontal diseases

Periodontitis is affecting 11% of the global population (Marcenes et al., 2013). Its incidence varies in different populations and depends on oral hygiene and socio-economic status (Rylev and Kilian, 2008). Periodontitis is a chronic infectious disease of the supporting tissues of teeth and can lead to loss of connective tissue attachment, alveolar bone resorption, increased mobility of teeth and subsequent teeth loss. The etiology and pathogenesis of periodontitis is multifactorial and includes periodontal pathogens, biofilm formation, host immune response, and genetic risk factors (Laine et al., 2012).

The production of proteolytic enzymes and the respiratory burst of neutrophils mediated by enzymes such as NADPH oxidase and myeloperoxidase lead to generation of ROS/RNS and induce oxidative stress (Nizam et al., 2014; Syndergaard et al., 2014). These mechanisms play a key role in the pathogenesis of periodontitis as summarized by Chapple and Matthews in their comprehensive review (Chapple and Matthews, 2007). Salivary myeloperoxidase was found to be higher in periodontitis patients (Meschiari et al., 2013). The most common parameters of oxidative damage in association to periodontal diseases are markers of lipid peroxidation. Tsai et al. found higher lipid peroxidation in saliva of chronic periodontitis patients in a case-control study. An important secondary finding was that lipid peroxidation in saliva and gingival crevicular fluid correlated positively (Tsai et al., 2005). The association between periodontal status and salivary TBARS was confirmed in our large cross-sectional study with 217 patients (Celec et al., 2005). In a preliminary part of this study we found no correlation between salivary and plasma TBARS and, thus, local intraoral production of TBARS was hypothesized (Celec et al., 2005). Other studies confirmed higher lipid peroxidation in patients with severe but not moderate periodontitis (Mashayekhi et al., 2005; Khalili and Biloklytska, 2008) suggesting that the association between periodontitis and salivary TBARS might not be linear (Dalai et al., 2013). More recently, male patients, but not women with chronic periodontitis showed higher concentrations of salivary TBARS compared to healthy controls (Banasová et al., 2015). This could be due to changes in the changes in salivary cytokines during the menstrual cycle (Becerik et al., 2010). However, menstrual cycle related changes in salivary TAC concentrations have also been described with lower TAC during the ovulation period (Kawamoto et al., 2012). In line with these findings are the results of unique study on pregnant women. It has been shown that pregnancy has an effect on several markers of oxidative stress and, more importantly, that the association between TBARS and periodontal status found in the general population could not be proved for pregnant women or women who gave birth recently (Gümüs et al., 2015). This is very likely related to the hormonal changes in pregnancy that have major impact on oral health and local immune status (Gürsoy et al., 2014).

MDA is the most studied product of lipid peroxidation. However, MDA is only one of many products formed during lipid peroxidation. TOS assay developed by Erel provides a possibility to measure additive effects of oxidants (Erel, 2005). Akalin et al. utilized TOS assay to measure oxidants in saliva of chronic periodontitis patients. Higher MDA and TOS levels were observed in saliva and also gingival crevicular fluid of chronic periodontitis patients (Akalin et al., 2007). The importance of lipid peroxidation in saliva was confirmed in another showing that the lipid peroxidation in saliva of patients who smoke and suffer from periodontitis is higher when compared to healthy probands (Guentsch et al., 2008). Increased oxidative damage of DNA, lipids and proteins were observed in periodontitis patients in a cross-sectional study (Su et al., 2009). In our cross-sectional study with more than 200 participants salivary TBARS, AOPP, AGEs, and TAC were assessed in relation to dental health, gender, and age (Celecová et al., 2013). We have found a strong association between TBARS and papillary bleeding index and confirmed the results from a previous study (Celec et al., 2005). Moreover, it was shown that this association is age independent. Only TBARS and no other analyzed markers of oxidative stress were associated with periodontitis (Celecová et al., 2013). When MDA was assessed using HPLC periodontitis patients had higher MDA in gingival crevicular fluid but not in saliva (Wei et al., 2010). These results are in accordance with our results showing that salivary TBARS other than MDA are associated with periodontitis (Celec et al., 2005). On the other hand, superoxide dismutase and TOS levels were higher in periodontitis patients in both, gingival crevicular fluid and saliva. In addition, 16 weeks of non-surgical treatment decreased lipid peroxidation. Significant positive correlations were observed between clinical indices and MDA, TOS, and superoxide dismutase levels in both oral fluids (Wei et al., 2010). Scaling and root planing resulted in an increase in TAC, uric acid, and glutathione peroxidase, but also in a decrease in superoxide dismutase activity (Novakovic et al., 2014). This finding has been further strengthened by mass spectrometry analysis of ions in patients with periodontitis where Cu, Zn, and Mn ions—all important for superoxide dismutase isoforms were lower in patients vs. controls. In line are the found higher concentrations of isoprostanes as a consequence of lipid peroxidation (Huang et al., 2014). Several authors have assumed that higher lipid peroxidation in saliva is associated with increased percentage of gingival crevicular fluid in the saliva of periodontitis patients (Akalin et al., 2007; Su et al., 2009; Wei et al., 2010). Lipid peroxidation products in saliva might arise from ROS/RNS production by neutrophils activated by periodontal pathogens (Chapple and Matthews, 2007) or from direct microbial production of ROS/RNS (Vlková and Celec, 2009). Especially DNA damage markers seem to be associated with the presence and abundance of specific periodontal pathogens (Almerich-Silla et al., 2015).

Salivary 8-OHdG as a marker of DNA damage was higher in periodontitis patients in comparison to controls (Novakovic et al., 2013) but not in gingivitis patients (Sezer et al., 2012). Higher concentrations of salivary 8-OHdG in periodontal diseases were found also in several other studies (Takane et al., 2002; Sawamoto et al., 2005; Canakçi et al., 2009; Su et al., 2009; Novakovic et al., 2013; Miricescu et al., 2014). Initial periodontal therapy led to a decrease in salivary 8-OHdG. Thus, 8-OHdG could become a useful biomarker for evaluating the efficacy of periodontal treatment and individual prognosis (Takane et al., 2002, 2005). In another study, however, these findings could not be confirmed (Dede et al., 2013). Some studies have found a correlation between salivary 8-OHdG and the presence of *Porphyromonas gingivalis* or the large mitochondrial DNA deletion in gingival tissues (Canakçi et al., 2009).

Oxidative damage to proteins may have dangerous consequences in a cell due to their important catalytic functions (Dalle-Donne et al., 2003). Protein carbonyl content is the most widely used marker of protein oxidation (Sezer et al., 2012). It is associated with clinical indices of oral health, especially if gender of the patients is taken into account (Sculley and Langley-Evans, 2003). This has been confirmed in another study focusing on the prognostic potential of specifically oxidized salivary proteins such as transferrin, human IgG1 heavy chain fragment and amylase (Su et al., 2009). AOPP was originally suggested as a marker of oxidative damage in plasma of chronic uremic patients (Witko-Sarsat et al., 1996). Only two studies coming from our group have analyzed AOPP in saliva in relation to periodontal status. However, we have found an association between salivary AOPP and papillary bleeding index neither in adults (Celecová et al., 2013) nor in children (Tóthová et al., 2013a). Oxidative and carbonyl stress are closely linked. Reactive carbonyl groups can non-enzymatically react with amino groups of proteins leading to the production of AGEs. Lipid peroxidation products including MDA possess reactive carbonyl groups and can substitute carbohydrates in the Maillard reaction as the basis of AGEs production (Miyata et al., 2000). Similarly to AOPP, salivary AGEs were not associated with periodontal status in any of the studied group of patients (Celecová et al., 2013; Tóthová et al., 2013a).

Relatively few studies examined the concentrations of salivary NO in patients with gingivitis or periodontitis. Most of them observed higher NO concentrations depending on the periodontitis severity when compared to healthy controls (Reher et al., 2007; Parwani et al., 2012; Sundar et al., 2013; Wadhwa et al., 2013; Poorsattar Bejeh-Mir et al., 2014), but NO was decreased by the treatment (Parwani et al., 2012). Interestingly, in another study nitrite concentrations were lowered by treatment only in erythrocytes and not in plasma or saliva (Meschiari et al., 2015). Salivary concentrations of NO can be altered by other factors, especially smoking. Some studies, however, found lower salivary NO in patients with periodontitis compared to the healthy controls (Aurer et al., 2001; Andrukhov et al., 2013). The reasons for these discrepancies might include differences in the analytical methods, but also in the pre-analytic phase.

Similar reasons could explain some of the contradictory findings on TAC (Chapple and Matthews, 2007). In most of the studies TAC was found to be lower in patients with periodontitis (Chapple, 1997; Diab-Ladki et al., 2003; Mashayekhi et al., 2005). This has been confirmed by another study, while the three main specific salivary antioxidants assessed—uric acid, ascorbic acid, and albumin were not significantly different from controls (Diab-Ladki et al., 2003). A study focusing on the antioxidant melatonin found that neither plasma, nor salivary concentrations were different between patients and controls. The difference with lower concentrations for patients was found in the gingival tissue showing that saliva is not representative for all analyzed markers in the oral cavity (Balaji et al., 2015). Mashayekhi et al. have shown that TAC depends on the clinical severity of periodontitis. In addition, the role of cyclic nucleotides cAMP and cGMP, which were lower in patients vs. controls was highlighted in this study (Mashayekhi et al., 2005). Two other studies, however, found no difference between patients and controls in salivary TAC (Brock et al., 2004; Tóthová et al., 2013b). In contrast, higher salivary TAC was observed in periodontitis patients in a cross-sectional study (Su et al., 2009). Patients with periodontitis in that study were, however, older and consumed higher amounts of antioxidants than controls. Nevertheless, after adjusting for age and antioxidant intake, the multivariate analysis showed positive correlation between salivary TAC and periodontitis (Su et al., 2009). The crucial difference might, thus, lie in the dynamics of the disease that could differ between studies and is rarely reported. There might be also an often overlooked sex difference in salivary TAC being lower in women (Sculley and Langley-Evans, 2003). Miricescu et al. have reported lower salivary TAC and also uric acid as well as glutathione peroxidase in whole saliva of patients with periodontitis (Novakovic et al., 2013). In other studies lower salivary glutathione—the main intracellular antioxidant and ceruloplasmin—an important extracellular antioxidant were reported in periodontitis patients in comparison to healthy probands (Tsai et al., 2005; Dalai et al., 2013). This could be explained by a depletion of the antioxidants due to increased ROS/RNS generation. It is unclear whether periodontal pathogens could be directly involved (Tsai et al., 2005). The studies on salivary oxidative stress and antioxidant status markers in relation to periodontal disease are summarized in Table 2.

**Table 2:** **Association studies investigating salivary markers of oxidative stress and periodontal diseases**.

**Patients**	**Analyzed markers and methods**	**Detected concentrations**	**Results**	**References**
36 CP patients, 28 controls	MDA (HPLC), TOS (SPH)	Control group, MDA (median 0.06 μM), TOS (4.16 ± 0.63 mM H_2_O_2_ Equivalent); CP group, MDA (median 0.1 μM), TOS (6.03 ± 1.37 mM H_2_O_2_ Equivalent)	↑ MDA and ↑ TOS in CP patients (*p* < 0.05); positive correlations was observed between periodontal parameters and MDA and TOS (*p* < 0.05)	Akalin et al., 2007
30 CP patients (15 smokers), 30 controls (15 smokers)	MDA (SFL), GSHPx (SPH), TAC (PCL) (baseline and 6 moth after non-surgical treatment)	Control group, non-smokers, MDA (0.065 ± 0.05 μmol/L), GSHPx (5.78 ± 3.77 U/L), TAC flow rate (0.52 ± 0.20 μmol/mL); Control group, smokers, MDA (0.085 ± 0.08 μmol/L), GSHPx (7.72 ± 2.70 U/L), TAC flow rate (0.75 ± 0.24 μmol/mL) CP group, non-smokers, MDA (0.095 ± 0.05 μmol/L), GSHPx (16.08 ± 13.34 U/L), TAC flow rate (0.37 ± 0.24 μmol/mL) CP group, smokers, MDA (0.123 ± 0.08 μmol/L), GSHPx (21.10 ± 18.65 U/L), TAC flow rate (0.29 ± 0.21 μmol/mL) Post-treatment, CP group, non-smokers, MDA (0.060 ± 0.09 μmol/L), GSHPx (6.54 ± 2.84 U/L), TAC flow rate (0.44 ± 0.22 μmol/mL) Post-treatment, CP group, smokers, MDA (0.060 ± 0.05 μmol/l), GSHPx (6.74 ± 4.33 U/l), TAC flow rate (0.42 ± 0.17 μmol/ml)	↑ MDA in smoking CP patients compared to non-smoking controls (*p* < 0.05);↑ GSHPx and ↓ TAC flow rate in CP patients (*p* < 0.05); non-surgical periodontal treatment lead to ↓ MDA and ↓ GSHPx (*p* < 0.05)	Guentsch et al., 2008
48 CP patients, 35 controls	MDA (HPLC), TOS (SPH), SOD (SPH) (baseline and 16 weeks after non-surgical treatment)	Control 1, MDA (0.10 ± 0.02 mM), TOS (6.75 ± 1.02 mM), SOD (174.9 ± 21.07 U/mg protein); Control 2, MDA (0.11 ± 0.03 mM), TOS (5.69 ± 1.03), SOD (177.6 ± 24.61 U/mg protein); CP 1 (before therapy), MDA (0.11 ± 0.05 mM), TOS (9.12 ± 1.77 mM), SOD (216.4 ± 36.78 U/mg protein); CP 2 (after therapy), MDA (0.09 ± 0.01 mM), TOS (5.61 ± 0.95 mM), SOD (169.8 ± 23.65 U/mg protein)	↑ TOS and ↑ SOD in CP group (*p* < 0.05); ↓ SOD and ↓ TOS after therapy (*p* < 0.05); positive correlations between clinical parameters and MDA, TOS and SOD levels	Wei et al., 2010
74 patients with periodontitis, 3 groups -early (*n* = 30), moderate (*n* = 30), severe (*n* = 14), 30 controls	MDA (SPH)	Control group, MDA (5.16 ± 0.03 μmol/mL) Early periodontitis group, MDA (28.08 ± 1.56 μmol/mL); Moderate periodontitis group, MDA (39.01 ± 1.59 μmol/mL); Severe periodontitis group, MDA (65.20 ± 2.00 μmol/mL)	Significant differences in MDA levels of patients with early, moderate and severe periodontitis in comparison to control patients (*p* < 0.05)	Khalili and Biloklytska, 2008
Preliminary study: 13 CP patients, 9 controls	GSH (SPH), GPx (SPH), LPO (SPH)	Preliminary stud Control group, GSH (606.67 ± 191.02 μmol/L), GPx (92.90 ± 58.58 mU/mL), LPO (0.13 ± 0.08 μmol/L);	↓ GSH (*p* < 0.05), ↑ lipid peroxidation (*p* < 0.0005) and no difference in GPx activity in patients than in controls; ↓ lipid peroxidation (*p* < 0.05), ↑ GSH (*p* < 0.001), and no change in GPx activity in patients after periodontal treatment	Tsai et al., 2005
Subsequent study: 22 CP patients	Subsequent study: (baseline and 1 month after initial periodontal treatment)	Periodontitis group, GSH (373.04 ± 287.42 μmol/L), GPx (92.99 ± 74.40 mU/mL), LPO (0.66 ± 0.36 μmol/L) Subsequent study: Periodontitis group before treatment, GSH (353.59 ± 141.93 μmol/L), GPx (96.50 ± 35.14 mU/mL), LPO (0.63 ± 0.49 μmol/L); Periodontitis group after treatment, GSH (602.92 ± 170.15 μmol/L), GPx (99.34 ± 45.72 mU/mL), LPO (0.41 ± 0.26 μmol/L)		
217 consecutive stomatologic patients	TBARS (SFL), MDA (HPLC)	TBARS (0.05–2.2 μmol/L^#^), MDA (0.3 μmol/mL^#^)	↑ TBARS tightly associated with ↑ PBI (adjusted for age and sex, *p* < 0.001)	Celec et al., 2005
204 consecutive stomatologic patients	TBARS (SPH), AOPP (SPH), TAC (SPH), AGEs (SFL)	Group with PBI = 0, TBARS (0.038 μmol/L^#^); Group with PBI = 1, TBARS (0.045 μmol/L^#^); Group with PBI = 2, TBARS (0.055 μmol/L^#^)	TBARS associated with PBI (*p* = 0.004); ↑ TBARS (*r* = 0.235, *P* = 0.004), ↑ AGEs (*r* = 0.141, *P* = 0.043) and ↑ TAC (*r* = 0.225, *P* = 0.002) with age	Celecová et al., 2013
82 consecutive pediatric dental patients	AOPP (SPH), TBARS (SFL), AGES (SFL), FRAP (SPH), TAC (SPH)	Children with PBI: 0, AOPP (140 μmol/L^#^), TBARS (0.029 μmol/L^#^), AGES (1 g/L^#^), FRAP (75 μmol/L^#^), TAC (1.6 mmol/L^#^); Children with PBI: 1, AOPP (125 μmol/L^#^), TBARS (0.038 μmol/L^#^), AGES (0.95 g/L^#^), FRAP (65 μmol/L^#^), TAC (1.65 mmol/L^#^); Children with PBI: 2, AOPP (150 μmol/L^#^), TBARS (0.056 μmol/L^#^), AGES (0.85 g/L^#^), FRAP (70 μmol/L^#^), TAC (1.5 mmol/L^#^)	variability of PBI explains 10.9% of the variance of TBARS (*p* = 0.02); TAC and FRAP were partially determined by PBI (16.9% and 7.9%, *p* < 0.05)	Tóthová et al., 2013a
29 periodontitis patients, 20 controls	8-HOdG (C-ELISA kit) (baseline and 2–4 month after initial periodontal treatment)	Control group, 8-HOdG (1.48 ± 0.08 ng/mL) Periodontitis group, 8-HOdG (4.36 ± 0.18 ng/mL) Before therapy, 8-HOdG (4.05 ± 0.17 ng/mL) After therapy, 8-HOdG (1.75 ± 0.09 ng/mL)	↑ 8-HOdG in periodontitis patients (*p* < 0.01); 8-OHdG correlated with P. gingivalis (*p* < 0.01); ↓ 8-OHdG after initial periodontal treatment (*p* < 0.01)	Sawamoto et al., 2005
24 patients with periodontitis, 3 groups—early (*n* = 8), moderate (*n* = 8), advanced (*n* = 8), 8 controls	TBARS (SPH), TAP (SPH), cAMP (ELISA), cGMP (ELISA)	Control group, TBARS (1.2 μmol/mL^#^), TAP (2 μmol/mL^#^), cAMP (32 pmol/mL^#^), cGMP (3.6 μmol/mL^#^) Early periodontitis group, TBARS (1.22 μmol/mL^#^), TAP (1.8 μmol/mL^#^), cAMP (27 pmol/mL^#^), cGMP (3.3 μmol/mL^#^); Moderate periodontitis group, TBARS (1.22 μmol/mL^#^), TAP (1.75 μmol/mL^#^), cAMP (23 pmol/mL^#^), cGMP (3.1 μmol/mL^#^); Advanced periodontitis group, TBARS (1.35 μmol/mL^#^), TAP (1.5 μmol/mL^#^), cAMP (25 pmol/mL^#^), cGMP (3 μmol/mL^#^)	↓ cAMP and ↓ cGMP in patients with moderate and advanced periodontitis (*p* < 0.01); ↑ TBARS (*p* < 0.01) and ↓ TAP (*p* < 0.01) in patients with advanced periodontitis	Mashayekhi et al., 2005
58 periodontitis patients, 234 controls	8-OHdG (ELISA), 8-epi-PGF2α (ELISA), total protein carbonyls (ELISA), TAC (SPH)	Control group, 8-OHdG (42.65 ng/ml), 8-epi-PGF2α (43.57 pg/ml), protein carbonyls (0.96 nmol/mg protein), TAC (0.46 mM); Periodontitis group, 8-OHdG(66.84 ng/ml), 8-epi-PGF2α (62.72 pg/ml), protein carbonyls (1.79 nmol/mg protein), TAC (0.71 mM)	↑ 8-OHdG (*p* = 0.0003), ↑ 8-epi-PGF2α (*p* = 0.0001), and ↑ carbonylated proteins (*p* < 0.0001) in periodontal patients; 8-OHdG, 8-epi-PGF2α, and carbonylated proteins independently negatively associated with CPITN (*P* = 0.004, 0.02, and 0.0001); a positive correlation between TAC and periodontal disease status (*p* < 0.0001)	Su et al., 2009
16 patients with stage I periodontitis, 16 patients with stage II periodontitis, 15 controls	MDA, ceruloplasmin (detailed methods not provided)	Control group, MDA (0.58 ± 0.14 nmol/mL), ceruloplasmin (3.46 ± 1.25 mg%); Stage I periodontitis, MDA (2.17 ± 0.55 nmol/mL), ceruloplasmin (2.14 ± 1.18 mg%); Stage II periodontitis, MDA (2.05 ± 0.48 nmol/mL), ceruloplasmin (1.11 ± 0.66 mg%)	↑ MDA in patients with stage I and stage II periodontitis in comparison to control (*p* < 0.001); ↓ MDA in patients with stage II periodontitis compared to the patients with stage I periodontitis (*p* > 0.1); ↓ ceruloplasmin in stage II periodontitis patients compared to control patients (*p* < 0.001)	Dalai et al., 2013
20 CP patients, 20 controls	8-HOdG (ELISA), MDA (SPH), uric acid (SPH), TAC (SPH), GPx (SPH)	Control group, 8-HOdG (6.46 ± 0.93 ng/mg albumin), MDA (0.25 ± 0.4 nmol/mg albumin), uric acid (3.12 ± 0.85 mg/mg albumin), TAC (1.24 ± 0.16 nmol/mg albumin), GPx (28.16 ± 11.95 U/mg albumin); CP group, 8-HOdG (6.78 ± 1.80 ng/mg albumin), MDA (0.296 ± 0.10 nmol/mg albumin), uric acid (2.41 ± 0.265 mg/mg albumin), TAC (0.75 ± 0.16 nmol/mg albumin), GPx (15.81 ± 7.22 U/mg albumin)	8-OHdG, MDA higher in CP group (*p* < 0.05); uric acid, TAC and GPx decreased in CP patients vs. controls (*p* < 0.05), oxidative stress markers associated with alveolar bone loss biomarkers	Miricescu et al., 2014
32 patients with periodontits, 32 control patients	8-OHdG (C-ELISA kit)	Control group, 8-OHdG (1.41 ± 0.22 ng/ml) CP group, 8-OHdG (3.76 ± 0.30 ng/ml)	↑ 8-OHdG in patients with periodontitis (*p* < 0.01); positive correlation between the occurrence of the 5-kbp mtDNA deletion and salivary 8-OHdG concentrations	Canakçi et al., 2009
78 patients with periodontitis, 17 controls	8-HOdG (ELISA)	Control group, 8-OHdG (1.56 ± 0.10 ng/mL); Periodontitis group, 8-OHdG (4.28 ± 0.10 ng/mL)	↑ 8-HOdG in patients with periodontitis (*p* < 0.01)	Takane et al., 2002
20 CP patients, 20 CG patients, 20 controls	8-HOdG (C-ELISA)	Control group, 8-OHdG (1.56 ± 0.12 ng/mL) CG group, 8-OHdG (1.58 ± 0.13 ng/mL) CP group, 8-OHdG (3.13 ± 0.22 ng/mL)	↑ 8-HOdG in CP than in CG and H groups (*p* < 0.001); correlation between the salivary 8-OHdG and CAL in patients with CAL > 3 mm (*p* < 0.001)	Sezer et al., 2012
34 CP patients, two groups- with periodontally involved teeth of hopeless prognosis (*n* = 16), without teeth of hopeless prognosis (*n* = 18), 17 controls	8-OHdG (C-ELISA) (baseline and 2–6 months after initial periodontal treatment)	Control group, 8-OHdG (1.56 ± 0.1 ng/mL) Before treatment: Group without periodontally hopeless teeth, 8-OHdG (2.35 ± 0.18 ng/mL); Group with periodontally hopeless teeth, 8-OHdG (4.78 ± 0.14 ng/mL); After treatment: Group without periodontally hopeless teeth, 8-OHdG (1.73 ± 0.16 ng/mL); Group with periodontally hopeless teeth, 8-OHdG (2.02 ± 0.31 ng/mL)	↑ 8-OHdG in those with than in subjects without periodontally-involved teeth of hopeless prognosis (*p* < 0.05) and healthy controls (*p* < 0.01); ↓ 8-OHdG levels after treatment of subjects with (*p* < 0.01) but not those without periodontally involved teeth of hopeless prognosis	Takane et al., 2005
24 patients with CP, 24 controls	8-OHdG (baseline and 10 days, 1 month, 3 months after initial periodontal therapy)	CP baseline 8-OHdG (605.5 ± 139.1) controls 8-OHdG (550.52 ± 150.28) CP 10 days 8-OHdG (543.1 ± 154.8) CP 1 month 8-OHdG (542.0 ± 154.6) CP 3 months 8-OHdG (534.3 ± 151.2)	salivary 8-OHdG did not differ between groups or during initial periodontal therapy (*p* > 0.05)	Dede et al., 2013
129 patients, cohort study	TAC (SPH), ascorbate (SPH), urate (SPH), albumin (SPH), protein carbonyl (SPH)	Men, protein carbonyl (8.46 ± 1.71 fmol/g of protein), TAC flow rate (0.31 ± 0.02 μmol/mL/min), ascorbate flow rate (4.27 ± 0.44 nmol/mL/min), albumin flow rate (5.44 ± 0.74 nmol/mL/min), urate flow rate (108.8 ± 12.0 nmol/mL/min); Women, protein carbonyl (19.26 ± 7.09 fmol/g of protein), TAC flow rate (0.19 ± 0.09 μmol/mL/min), ascorbate flow rate (2.88 ± 0.30 nmol/mL/min), albumin flow rate (4.03 ± 0.74 nmol/mL/min), urate flow rate (58.7 ± 6.6 nmol/mL/min);	↓ TAC in women than in men (*p* < 0.01), protein carbonyls 2.3 times ↑ in women than in men (*p* = ns); TAC corrected for sex ↑ in severe disease group (*p* < 0.05), protein carbonyls corrected for sex ↓ in severe disease group (*p* < 0.05);association between protein carbonyl concentration and low CPITN score (*p* = 0.018) and female sex (*p* = 0.020); antioxidant status and risk of disease (classified as CPITN < 18) was significantly related to TAC flow rate (*p* = 0.043)	Sculley and Langley-Evans, 2003
17 patients with severe periodontitis, 20 controls	Uric acid (SPH), albumin (SPH), ascorbic acid (SPH), TAC (SPH)	Control group, uric acid (160 μmol/L^#^), albumin (14 μmol/L^#^), ascorbic acid (9 μmol/L^#^), TAC (340 μmol/L^#^); Periodontitis group, uric acid (140 μmol/L^#^), albumin (11 μmol/L^#^), ascorbic acid (7 μmol/L^#^), TAC (220 μmol/L^#^)	↓ TAC in patients with periodntitis (*p* < 0.05); uric acid, ascorbic acid, and albumin are not significantly affected (*p* = ns)	Diab-Ladki et al., 2003
20 CP patients, 20 controls	TAC (ECL)	Control group, TAC (0.14 ± 0.06 nmoles/30 s sample) CP group, TAC (0.18 ± 0.08 nmoles/30 s sample)	↓TAC in CP group than in control group (*p* = ns)	Brock et al., 2004
18 patients with periodontitis, 16 controls	TAC (ECL)	Control group, TAC (254 ± 110 mumol/L); Peridontitis group, TAC (175 ± 53 mumol/L)	↓ TAC in the peridontitis patients than in control patients (*p* < 0.01)	Chapple et al., 1997
24 patients with gingivitis, 23 with periodontitis, and 23 controls	SOD (SPH), thiol antioxidant concentrations (SPH) (baseline and 15 days after non-surgical treatment)	Before treatment: Control group, SOD (129.93 ± 3.98 U/0.5 mL), thiol (36.99 ± 2.14 μmol/L) Gingivitis group, SOD (88.28 ± 2.56 U/0.5 mL), thiol (30.15 ± 3.13 μmol/L) Periodontitis group, SOD (39.99 ± 3.52 U/0.5 mL), thiol (15.09 ± 2.26 μmol/L) After treatment: Control group, SOD (158.69 ± 3.61 U/0.5 mL), thiol (53.96 ± 1.72 μmol/L) Gingivitis group, SOD (133.56 ± 2.16 U/0.5 mL), thiol (45.83 ± 2.09 μmol/L) Periodontitis group, SOD (61.44 ± 2.67 U/0.5 mL), thiol (27.41 ± 1.88 μmol/L)	↑ SOD and thiol concentrations post-treatment in all the three groups, comparison between the three groups post-treatment did not show any significant difference in improvement of superoxide dismutase or thiol concentrations	Karim et al., 2012
21 CP patients	GPx, SOD, albumin, uric acid, total antioxidative status (TAS) (before and after non-surgical treatment)	N/A	↑ uric acid, albumin, GPx, TAS; ↓ SOD activity after treatment; correlation between GPx and plaque index, SOD and gingival index before therapy; correlation between SOD and bleeding on probing, and TAS and bleeding on probing after therapy	Novakovic et al., 2013
23 CP patients (14 females, 9 males)	TBARS, AGEs (SFL), TAC FRAP (SPH)	N/A	↑ TBARS in male CP (*p* < 0.01); ↓ TAC in female CP (*p* < 0.001)	Banasová et al., 2015
19 controls (8 females, 11 males)				
30 CP patients 30 controls	MDA, SOD, GR, CAT (all markers SPH)	CP group MDA (9.34 ± 8.15 nmol/ml), SOD (19.76 ± 11.53 U/ml), CAT (0.08 ± 0.13 U/min/mg prot), GR (12.51 ± 6.39 U/min/mg prot); Control group MDA (1.39 ± 1.28 nmol/ml), SOD (30.22 ± 7.03 U/ml), CAT (0.37 ± 0.28 U/min/mg prot), GR (25.50 ± 9.11 U/min/mg prot)	↑ MDA in CP; ↓ SOD, CAT and GR in CP compared to control group Positive correlation between MDA and periodontal status; Negative correlation between SOD, CAT and GR and periodontal status	Trivedi et al., 2015
30 CP patients with diabetes mellitus type 2	SOD GR	CP group MDA (9.09 ± 8.16 nmol/ml) SOD (19.93 ± 12.05 U/ml)	↑ MDA in CP irrespective of diabetes;	Trivedi et al., 2014
30 CP patients without systemic disease	CAT MDA (all markers SPH)	GR (13.63 ± 6.46 U/min/mg prot) CAT (0.08 ± 0.14 U/min/mg prot)	SOD and GR differed in CP patients with diabetes mellitus type 2 compared to CP without systemic disease	
30 controls with diabetes mellitus type 2 30 healthy controls		CP group with DM type 2 MDA (10.79 ± 8.07 nmol/ml) SOD (14.08 nmol/ml 4.28 U/ml) GR (18.33 ± 7.47 U/min/mg prot) CAT (0.04 ± 0.03 U/min/mg prot) controls without systemic disease MDA (1.53 ± 1.30 nmol/ml) SOD (29.64 ± 6.98 U/ml) GR (24.57 ± 7.97 U/min/mg prot) CAT (0.38 ± 0.29 U/min/mg prot) Controls with DM type 2 MDA (1.91 ± 1.72 nmol/ml) SOD (13.45 ± 2.80 U/ml) GR (13.73 ± 2.79 U/min/mg prot) CAT (0.04 ± 0.04 U/min/mg prot)		
33 CP patients 35 patients with generalized aggressive periodontitis (GAP) 30 healthy controls	MDA (HPLC) TOS TAC (SPH)	CP group MDA 0.15 μmol/L (0.1 to 0.18), TOS 6.32 μmol/L (5.51 to 7), TAC (0.53 ± 0.11 mmol/L); GAP group MDA 0.15 μmol/L (0.14 to 0.18), TOS 7.80 μmol/L (7.5 to 8.5), TAC (7.80 ± 0.08 mmol/L); Control group MDA 0.08 μmol/L (0.06 to 0.12), TOS 4.22 μmol/L (3.75 to 4.53), TAC (4.22 ± 0.08 mmol/L)	↓ TAC ↑ MDA, TOS and oxidation stress index in CP and GAP in comparison with controls	Baltacioglu et al., 2014
47 CP patients (24 smokers, 23 non-smokers)	8-OHdG, 4-HNE, GPx (ELISA)	Smokers with CP 8-OHdG (8.02 ng/ml ± 1.46), 4-HNE (144.28 pg/ml ± 59.18), GPx (36.81 U/ml ± 9.16) Non-smokers with CP 8-OHdG (7.85 ng/ml ± 1.74), 4-HNE (133.09 pg/ml ± 61.92), GPx (30.59 U/ml ± 15.06)	↑ 8-OHdG in patients with CP (irrespective of smoking) compared to controls; ↓ 8-OHdG after treatment	Hendek et al., 2015
46 controls (23 smokers, 23 non-smokers)		Smokers controls 8-OHdG (7.50 ng/ml ± 1.46), 4-HNE (131.40 pg/ml ± 23.03), GPx (29.22 U/ml ± 16.45) Non-smokers controls 8-OHdG (6.74 ng/ml ± 1.96), 4-HNE (130.36 pg/ml ± 21.09), GPx (24.36 U/ml ± 13.28)		
30 CP (baseline, 1 month after periodontal treatment) 30 controls	8-OHdG (ELISA)	8-OHdG CP group (645.18 pg/ml ± 84.91), control group (527.23 pg/ml ± 62.19) after treatment 8-OHdG CP group (532.18 pg/ml ± 91.37)	↑ 8-OHdG in patients with CP; ↓ 8-OHdG after treatment	Arunachalam et al., 2015
23 CP (8 females, 15 males) 25 controls (15 females, 10 males); (baseline, 6 weeks after periodontal treatment)	8-OHdG (ELISA and LC-MS/MS)	N/A	↓ 8-OHdG after treatment; correlation between plaque index, gingival index, probing pocke depth, clinical attachment level, bleeding on probing	Kurgan et al., 2015
31 CP patients baseline and after periodontal treatment	TAC (SPH)	TAC before treatment (0.655 μmol/L ± 0.281) TAC after treatment (0.962 μmol/L ± 0.287)	↑ TAC after periodontal treatment	Shirzaiy et al., 2014
CP patient aggressive periodontitis patients controls (*n* = 20/per group) aged 25–55; both genders	NO (SPH)	CP NO (16.53 ± 1.51) Aggressive periodontitis NO (16.39 ± 2.38) Control NO (5.69 ± 0.93)	↑ NO in both periodontal groups compared to controls positive correlation of NO and periodontal parameters in both groups of patients	Sundar et al., 2013
89 generalized severe CP patients, 56 healthy controls (non-smokers); both gender	NO metabolites (SPH)	Only graphs available	↓ NO in CP compared to healthy controls	Andrukhov et al., 2013
CP patients divided into 2 groups according smoking status and healthy controls (*n* = 20/per group)	NO (SPH)	Non-smoking CP NO (79.52 μmol/L ± 24.88) Smoking CP NO (153.84 μmol/L ± 44.04) Controls NO (27.70 μmol/L ± 8.04)	↑ NO in both periodontal groups compared to controls; smoking significantly effects NO levels	Wadhwa et al., 2013
Patients with gingivitis periodontits and controls (*n* = 14/per group); age (30–50 years); both gender	Nitrite Nitrate Total NO (ELISA)	Gingivitis group nitrite (79.64 ± 4.62) Nitrate (79.36 ± 4.62) Total NO (159 ± 9.25) CP group nitrite (173.26 ± 9.26) Nitrate (172.99 ± 9.26) Total NO (346.25 ± 18.52) Control group nitrite (33.19 ± 4.69) Nitrate (50.39 ± 17.79) Total NO (100.95 ± 35.60)	↑ NO in gingivitis and periodontitis patients compared to controls	Poorsattar Bejeh-Mir et al., 2014
Patients with gingivitis periodontits and controls (*n* = 30/per group)	NO (SPH)	Gingivitis group NO pretreatment (430.60 ± 67.97) NO postreatment (269.07 ± 53.08) CP group NO pretreatment (537.67 ± 80.06) NO postreatment (326.73 ± 41.03) Control group NO pretreatment (241.10 ± 83.72)	↑ NO in gingivitis and periodontitis patients compared to controls	Parwani et al., 2012
Rapidly progressive periodontitis, adult periodontitis and healthy controls (*n* = 25/per group)	NO_2_(SPH)	Progressive periodontitis group NO_2_(2.5 ± 3.27 μmol/l) Adult periodontitis group NO_2_(11.1 ± 8.23 μmol/l) Control group NO_2_(22.4 ± 17.04 μmol/l)	↓ NO_2_ in CP patients compared to healthy controls; patiemts with rapidly progresive perriodontitis had ↓ NO_2_compared to patients with adult form of periodontitis	Aurer et al., 2001

CP, chronic periodontitis; CG, chronic gingivitis; TAC, total antioxidant capacity; TAP, total antioxidant power; AOPP, advanced oxidation protein products; TBARS, thiobarbituric acid reacting substances; AGES, advanced glycation end products; FRAP, ferric reducing antioxidant power; 8-HOdG, 8-hydroxydeoxyguanosine; 8-epi PGF2α, 8-epi prostaglandin F2α; GPx, glutathione peroxidase; GSH, reduced glutathione; GR, glutathione reductase; MDA, malodialdehyde; LPO, lipid peroxidation; TOS, total oxidant status; SOD, superoxid dismutase; SPH, spectrophotometry; SFL, spectrofluorometry; PCL, photochemiluminescence; ECL, enhanced chemiluminescent assay; HPLC, high performance liquid chromatography; C-ELISA, competitive ELISA;

# *denotes levels estimated from graph*.

## Issues and limitations

**Figure 1:**
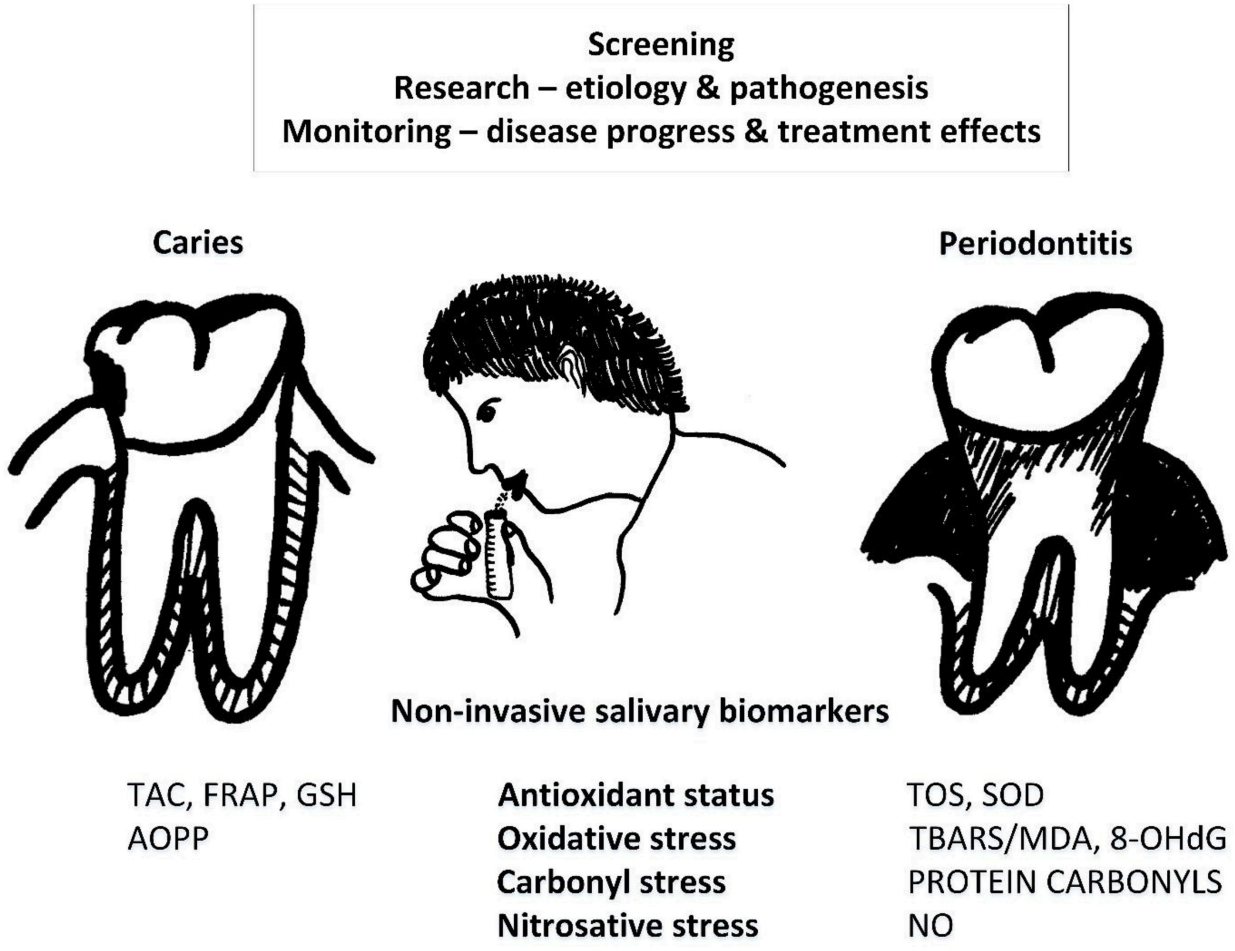
**Potential for non-invasive use of salivary biomarkers associated with caries and periodontitis**. Saliva can be used for disease screening, monitoring of progress, and treatment as well as in basic research on etiology or pathogenesis. 8-OHdG, 8-hydroxyguanosine; AOPP, advanced oxidation protein products; FRAP, ferric reducing antioxidant power; NO, nitric oxide; TAC, total antioxidant capacity; TBARS/MDA, thiobarbituric acid reacting substances/malondialdehyde; TOS, total oxidant status; SOD, superoxide dismutase.

Numerous studies summarized in this review show the potential of salivary markers of oxidative, carbonyl stress and antioxidant status as they seem to be associated with oral diseases, their severity and respond to treatment (Figure 1). However, there are several limitations that explain why these markers are not in routine clinical use yet. From the summary tables it is clear that the reported concentrations of the salivary markers differ by orders of magnitude between studies. With such a variability, contradictory results from the studies are not surprising. The reasons for such a variability are both, technical and biological. Various different methods and protocols for each particular assay are used as comprehensively reviewed by others (Palmieri and Sblendorio, 2007a; Wang et al., 2015a). Our research uncovered several pre-analytical factors that affect the salivary markers of oxidative stress including saliva collection, daytime, intake of antioxidants, tooth-brushing and others (Kamodyová and Celec, 2011; Kamodyová et al., 2013). Importantly, salivation should not be induced as such samples are biased in comparison to spontaneous salivation. And although collection devices such as Salivette make saliva collection even more effective, their use should be limited to biomarkers that are resistant to such a collection method. This is not the case for most of the studied markers of oxidative stress. Our unpublished preliminary data show that a major impact on the subsequent analyses can come from such a small step as centrifugation after saliva collection. Although our data do not support this assumption, it can be expected that prolonged storage at different temperatures can lead to variable results in the analysis. The stability of the biomarkers very likely differs from marker to marker.

Beyond periodontitis and caries there are other oral and systemic diseases that were studied in relation to salivary markers of oxidative stress. Oral precanceroses such as lichen planus and leukoplakia but also patients with oral squamous cell carcinoma were found to be associated with higher MDA (Lopez-Jornet et al., 2014; Metgud and Bajaj, 2014). In one study autistic children had lower salivary TAC in comparison to their healthy siblings, although this difference can be attributed to worse oral hygiene (Rai et al., 2012). Markers of carbonyl stress such as salivary AGEs are modified by the treatment in sleep apnea syndrome (Celec et al., 2012). Even patients with the Down syndrome have higher salivary MDA and superoxide dismutase when compared with controls (de Sousa et al., 2015). It is very likely that other physiological and pathological factors including renal diseases, diabetes mellitus, and systemic inflammatory disorders influence salivary concentrations of biomarkers and a lot of research is yet to be conducted. A summary of the current research on salivary oxidative stress markers including studies on extra-oral or systemic diseases was published recently (Buczko et al., 2015).

The level of evidence varies between the studies. Only few of them report results from more than 100 patients. With such high technical and biological variability it is clear that the number of patients/samples has to be high to extract meaningful information from the gathered data. The most important limitations are, however, the unclear causality of the observed associations. Even tight correlation do not have to be due to a causal relationship. The causality can only be tested in interventional experiments in animal models or human patients.

## Future outlook

**Figure 2:**
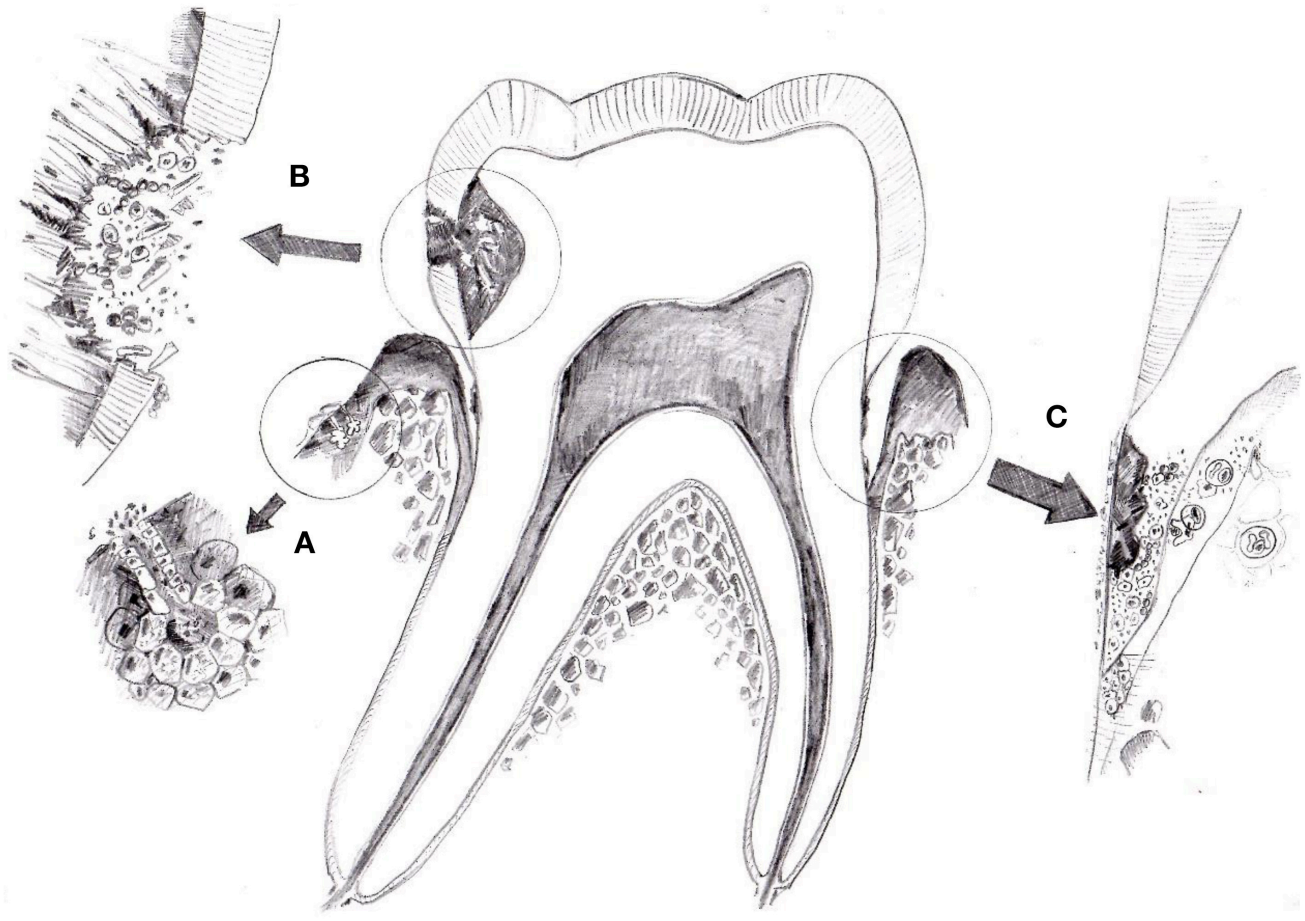
**The origin of salivary markers of oxidative stress**. Three possible sources are shown—blood plasma **(A)**, oral bacteria **(B)**, and immune cells **(C)**. Although direct evidence is lacking, salivary markers of oxidative stress seem to be of local oral origin. At least in periodontitis the production of reactive oxygen species by oral bacteria or activated neutrophils seems to be of importance. In systemic diseases diffusion from plasma could be the main source of salivary markers of oxidative stress.

Especially, the easy and non-invasive collection of saliva makes it a very interesting diagnostic fluid. Although numerous pitfalls have to be taken into account, analysis of salivary biomarkers could make it into routine clinic when it comes to salivary steroids, DNA, RNA, or the oral microflora. It is possible that in the future also salivary markers of oxidative stress will be used for screening and monitoring of oral diseases such as periodontitis or caries as these markers seem to be mostly of local oral origin (Figure 2). It should not be seen as a substitute for proper clinical examination but rather an adjuvant tool for various applications such monitoring of the patients with poor adherence to dental visits. Especially, if the biochemical detection methods will be developed into simple strips that change color based on the concentration of the particular marker similarly to urine strips. Such first example already exist with a strip detecting thiol compounds known to be higher in periodontitis patients (Khocht et al., 2013). However, before that numerous mentioned obstacles have to be solved. Future studies should concentrate on identification of the sources of the observed variability, widening the palette of the available markers and experiments proving the role of oxidative stress in the pathogenesis of oral diseases. Inflammatory markers measured in saliva are very informative regarding the periodontal status of patients (Rathnayake et al., 2013; Salminen et al., 2014). Cooperative efforts focused on large multi-centric studies with sufficient power and standardized analysis are needed. Promising targeted antioxidant treatments could be a way how to approach cases of therapy-resistant periodontitis as partially shown for melatonin (Kara et al., 2013; Arabaci et al., 2015). The potential of non-invasive diagnosis and treatment of oral diseases makes salivary markers of oxidative stress very attractive for further research. And for this future a paradigm shift will be needed as the research will very likely by dominated by high-throughput approaches such as salivary metabolome analysis (Dame et al., 2015).

### Conflict of interest statement

The authors declare that the research was conducted in the absence of any commercial or financial relationships that could be construed as a potential conflict of interest.
